# Pericardite Recorrente: Manifestação de Inflamação Sistêmica?

**DOI:** 10.36660/abc.20260127

**Published:** 2026-03-26

**Authors:** Marcio Roberto Moraes Carvalho

**Affiliations:** 1 Universidade Federal Fluminense Niterói RJ Brasil Universidade Federal Fluminense, Niterói, RJ – Brasil

**Keywords:** Pericardite, Recidiva, Inflamação Sistêmica

A pericardite recorrente representa uma das manifestações mais desafiadoras das doenças pericárdicas, em razão do impacto na qualidade de vida, do uso prolongado de terapias anti-inflamatórias e do risco de evolução para refratariedade.^1,2^ Embora classicamente descrita como condição episódica, evidências indicam que, em parcela significativa dos casos, a recorrência pode refletir persistência de inflamação sistêmica subjacente, frequentemente de natureza autoinflamatória, não plenamente capturada por marcadores inflamatórios convencionais.^2,3^ Nesse contexto, a pericardite recorrente evoluiu de abordagem local para entidade sistêmica; a ativação do inflamassoma e da interleucina-1 sustenta fenótipos mais graves e recorrentes.^3–5^

Índice de Inflamação Imune Sistêmica (*systemic immune-inflammation index, SII*), calculado como SII = (plaquetas × neutrófilos) / linfócitos, integra componentes centrais da inflamação sistêmica e da resposta imune, refletindo o equilíbrio entre imunidade inata, adaptativa e ativação plaquetária.^6–8^ Distingue-se de scores diagnósticos compostos, como o INFLA-score. O SII constitui um biomarcador quantitativo contínuo, destinado à estimativa da carga inflamatória sistêmica subjacente e ao monitoramento temporal da atividade imuno-inflamatória.^6–8^

Em doenças autoimunes, o SII se correlaciona de forma consistente com atividade da doença, resposta terapêutica e prognóstico. Na artrite reumatoide, se associa à inflamação e à redução dos níveis séricos de Klotho.^7^ Em espondilite anquilosante, artrite psoriásica e lúpus eritematoso sistêmico, se relaciona a escores de atividade, achados de imagem e desfechos, podendo superar a proteína C-reativa (PCR) e a velocidade de hemossedimentação.^8^

À luz desse arcabouço, a aplicação do SII à pericardite aguda recorrente se mostra conceitualmente consistente. No estudo de Unkun e Sengör,^9^ o SII demonstrou valor preditivo independente para recorrência após um primeiro episódio de pericardite aguda idiopática, mantendo associação robusta mesmo após ajuste para a PCR e superando marcadores hematológicos isolados.^10^ Esses achados reforçam a interpretação da recorrência pericárdica como manifestação clínica de inflamação sistêmica persistente, sustentada por desregulação imune, ativação do inflamassoma e liberação contínua de citocinas pró-inflamatórias.^3–5^

Sob essa perspectiva, a pericardite recorrente pode ser compreendida não apenas como um processo inflamatório localizado, mas como a expressão clínica de um pericárdio biologicamente ativo, que atua como sensor anátomo-histológico da atividade inflamatória mononuclear sistêmica.^2,3^ O pericárdio constitui um microambiente imunológico dinâmico, no qual interagem componentes da imunidade inata e adaptativa, com participação relevante tanto da inflamação macrofágica quanto linfocítica.

Essa heterogeneidade biológica ajuda a explicar a variabilidade clínica observada entre pacientes, incluindo maior risco de recorrência, dependência de corticosteroides e refratariedade terapêutica em fenótipos inflamatórios persistentes.^3,4^ Nesse cenário, se torna evidente a necessidade de ferramentas capazes de captar de forma integrada a atividade inflamatória subjacente.

Modelos prognósticos baseados em inteligência artificial emergem como ferramentas promissoras para integrar dados clínicos, terapêuticos e de imagem na estratificação de risco da pericardite recorrente. O modelo proposto por Yesilyaprak et al.^11^ representa avanço metodológico relevante, embora os próprios autores reconheçam como limitação a ausência de marcadores biológicos dinâmicos de inflamação sistêmica, apontando sua incorporação como via natural de refinamento futuro. Nesse contexto, biomarcadores integrativos como o SII assumem papel estratégico, permitindo não apenas a estimativa do risco clínico, mas a identificação precoce de inflamação sistêmica persistente, antes da consolidação de um curso refratário, avançando em direção a uma estratificação mecanística e personalizada.^9–11^

Em última análise, compreender a pericardite recorrente implica reconhecer que o pericárdio não apenas sofre a inflamação, mas também a sinaliza, e que a incorporação de biomarcadores sistêmicos integrativos, associada a modelos preditivos contemporâneos, pode ampliar a capacidade de estratificação biológica precoce.
