# Supplementary material for: Pericardite Recorrente: Manifestação de Inflamação Sistêmica?
Source: Arq Bras Cardiol. 2026 Mar 26;123(2):20260127. [Article in Portuguese] doi: 10.36660/abc.20260127 (PMC13128204; doi:10.36660/abc.20260127)
Supplement: Translation

# Translation: Recurrent Pericarditis: A Manifestation of Systemic Inflammation?

**DOI:** 10.36660/abc.20260127i

**Authors:** Marcio Roberto Moraes Carvalho

**Affiliations:** 1 Niterói RJ Brazil Universidade Federal Fluminense, Niterói, RJ – Brazil

**Keywords:** Pericarditis, Recurrence, Systemic inflammation

Recurrent pericarditis represents one of the most challenging manifestations of pericardial diseases, due to its impact on quality of life, the prolonged use of anti-inflammatory therapies, and the risk of progression to refractoriness.^1,2^ Although classically described as an episodic condition, evidence indicates that, in a significant proportion of cases, recurrence may reflect persistent underlying systemic inflammation, often of an autoinflammatory nature, not fully captured by conventional inflammatory markers.^2,3^ In this context, recurrent pericarditis has evolved from a local approach to a systemic entity; inflammasome and interleukin-1 activation support more severe and recurrent phenotypes.^3–5^

The Systemic Immune-Inflammation Index (SII), calculated as SII = (platelets × neutrophils)/lymphocytes, integrates central components of systemic inflammation and the immune response, reflecting the balance between innate and adaptive immunity and platelet activation.^6–8^ It differs from composite diagnostic scores, such as the INFLA-score. The SII constitutes a continuous quantitative biomarker, intended for estimating the underlying systemic inflammatory burden and for temporal monitoring of immuno-inflammatory activity.^6–8^

In autoimmune diseases, SII consistently correlates with disease activity, therapeutic response, and prognosis. In rheumatoid arthritis, it is associated with inflammation and reduced serum Klotho levels.^7^ In ankylosing spondylitis, psoriatic arthritis, and systemic lupus erythematosus, it is related to activity scores, imaging findings, and outcomes, and may outperform C-reactive protein (CRP) and erythrocyte sedimentation rate.^8^

In light of this framework, the application of SII to recurrent acute pericarditis appears conceptually consistent. In the study by Unkun and Sengör,^9^ SII demonstrated independent predictive value for recurrence after a first episode of idiopathic acute pericarditis, maintaining a robust association even after adjusting for CRP and outperforming isolated hematological markers.^10^ These findings reinforce the interpretation of pericardial recurrence as a clinical manifestation of persistent systemic inflammation, sustained by immune dysregulation, inflammasome activation, and continuous release of pro-inflammatory cytokines.^3–5^

From this perspective, recurrent pericarditis can be understood not only as a localized inflammatory process, but as the clinical expression of a biologically active pericardium, which acts as an anatomical-histological sensor of systemic mononuclear inflammatory activity.^2,3^ The pericardium constitutes a dynamic immunological microenvironment in which components of innate and adaptive immunity interact, with relevant participation of both macrophage and lymphocytic inflammation.

This biological heterogeneity helps explain the clinical variability observed among patients, including a higher risk of recurrence, corticosteroid dependence, and therapeutic refractoriness in persistent inflammatory phenotypes.^3,4^ In this scenario, the need for tools capable of capturing the underlying inflammatory activity in an integrated way becomes evident.

Artificial intelligence based prognostic models are emerging as promising tools for integrating clinical, therapeutic, and imaging data in the risk stratification of recurrent pericarditis. The model proposed by Yesilyaprak et al.^11^ represents a relevant methodological advance, although the authors themselves acknowledge the absence of dynamic biological markers of systemic inflammation as a limitation, pointing to their incorporation as a natural avenue for future refinement. In this context, integrative biomarkers such as SII assume a strategic role, allowing not only the estimation of clinical risk but also the early identification of persistent systemic inflammation, before the consolidation of a refractory course, advancing towards a mechanistic and personalized stratification.^9–11^

Ultimately, understanding recurrent pericarditis implies recognizing that the pericardium not only undergoes inflammation but also signals it, and that the incorporation of integrative systemic biomarkers, associated with contemporary predictive models, can enhance the capacity for early biological stratification.
